# A dataset of high-resolution digital elevation models of the Skeiðarársandur kettle holes, Southern Iceland

**DOI:** 10.1038/s41597-024-03515-6

**Published:** 2024-06-21

**Authors:** Joanna Ewa Szafraniec

**Affiliations:** University of Silesia in Katowice, Faculty of Natural Sciences, Institute of Earth Sciences, Będzińska 60, 41-200 Sosnowiec, Poland

**Keywords:** Geomorphology, Geodynamics, Hydrology

## Abstract

In studies of the relief evolution of smaller landforms, up to several dozen meters in width/diameter, digital elevation models (DEMs) freely accessible in different repositories may be insufficient in terms of resolution. Existing geophysical or photogrammetric equipment is not always available due to costs, conditions and regulations, especially for students or young researchers. An alternative may be the handy-held ground-based Structure from Motion technique. It allows us to obtain free high-resolution DEMs (~0.05 m) using open-source software. The method was tested on kettle holes of the glacial flood origin on Skeiðarársandur (S Iceland). The material was collected in 2022 at two outwash levels of different ages and vegetation cover. The dataset is available in the Zenodo repository; the first part is data processed in point clouds and DEMs, and the second includes original videos in MOV format. The data can be used as a reference to assess changes in the kettle hole relief in subsequent research seasons, as a methodological study for other projects, or for didactic purposes.

## Background & Summary

One of the cheaper and faster methods of obtaining information about the terrain through a digital elevation model (DEM) is the Structure from Motion (SfM) photogrammetric technique, “…developed since the 1980s into a valuable tool for generating 3D models from 2D imagery…”^1^. It is based on a combination of image recognition techniques (computer vision) and stereoscopic perception (visual perception), where it is essential to obtain at least 60% coverage of neighbouring photos^2^. The original algorithm was introduced by Ullman^3^, who stated that a three-dimensional image could be obtained from just four points visible from at least three directions. The most important factor was knowing the points’ exact location and the object’s assumed rigidity when changing direction. The more complex the shape, the more points are needed to describe it and, therefore, the more calculations.

Progress in this technique occurred with software development, including free software, which automated the calculations of scene geometry, camera position and orientation^4–8^. The final position of the bundle adjustment is determined by minimizing the errors of the sum of squares of the reprojection, i.e., the least squares method^9^. The process allows us to obtain an accurate 3D point cloud. The accuracy of the DEMs based on point clouds will depend on the terrain resolution, image quality and distortion, camera calibration, occurrence of vegetation, land surface features and the number, distribution and accuracy of ground control points (GCPs)^10^. We can add such apparent factors as meteorological conditions and user experience at each processing stage.

In recent years, there has been a marked increase in interest in the SfM technique. This increase is also noticeable in the number of publications^11^. In the years 2018–2022, the Web of Science indicates the number of publications in the range of 564–651 per year (phrases “structure from motion” and “structure-from-motion”). A review of the literature on applying SfM in geosciences can be found in some publications^1,2,11–13^. In the case of geomorphology, there is also a wide range of applications in studies, e.g. of river valleys and channel systems^14–21^, coasts^22–30^, weathering and slope processes^31–34^, soil erosion^35–37^, glacial and periglacial relief^13,38–41^, volcanic relief^42^, the role of macrofauna in the transformation of mudflat relief^43^, fault zone transformation and channel development^44^, the transformation of anthropogenic relief^45^.

Various platforms are used to obtain SfM photos, from masts or poles, through blimps, fixed-wing unmanned aerial systems (UAVs)/multicopters, kites to heli-/gyrocopters and light aircraft^1^. The use of UAVs is of particular interest^10,13,17,19,22,24,26,40,44,46–48^. However, most of the mentioned methods are still relatively expensive, and operation depends on, for example, battery life, wind conditions, permits, and authorizations. We can witness interesting, dynamic processes, but we do not always have access to expensive equipment, and the weather is not always favourable for carrying out research using them. The solution in such a situation, especially for small landforms or objects, may be using the ground-based SfM method. Although limited by image swath^1^, it is cost-free and gives satisfactory accuracy, guaranteeing promising future DEM resolution of centimetres. Research costs may be an essential issue for young researchers or students.

**Fig. 1:**
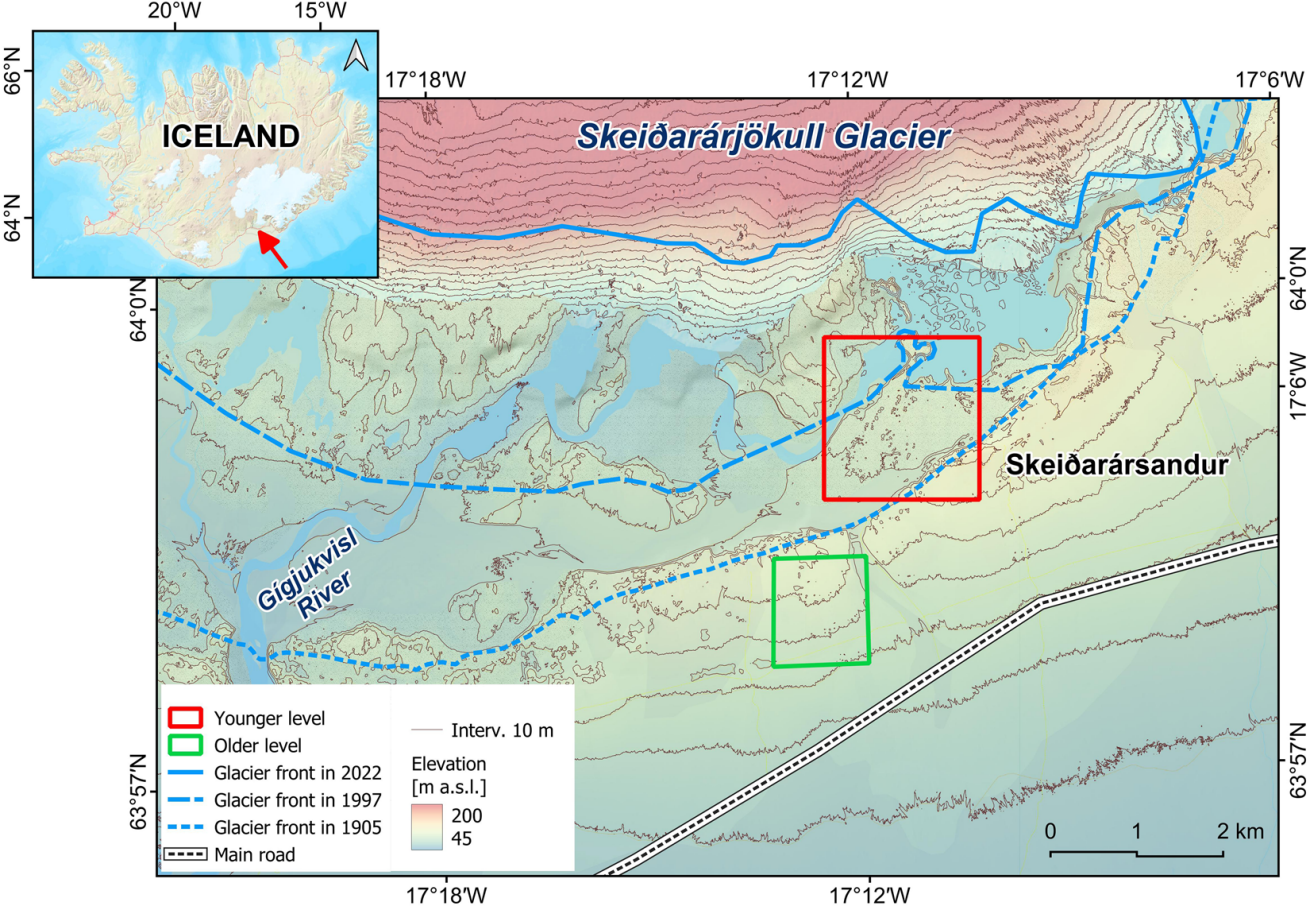
Skeiðarársandur, Southern Iceland – an area of the fieldwork research (based on ÍslandsDEM v. 1.0, 2 × 2 m, files 61 and 62, Landmælingar Íslands, https://dem.lmi.is/mapview/?application=DEM; 40% of the layer opacity; background and an insert map: wms layer LMI_Kort, Landmælingar Íslands; https://gatt.lmi.is/geonetwork/srv/eng/catalog.search#/metadata/35b42e6c-cff0-4c84-acdb-def228a5cbbc). The ISN93/Lambert 1993 coordinate system is used (the WGS84 coordinates notation).

The object of the study was closed depressions of glacial flood origin, called kettle holes, located on Skeiðarársandur in S Iceland, the most extensive European active outwash plain (Fig. 1). Their formation was related to the disintegration of buried glacial ice, detached from the glacier front when meltwaters burst into the forefield^41,49–56^. Further shaping depends on processes related to mass movements, water runoff, aeolian sedimentation and denudation, processes of colonization and plant succession.

The study aimed to obtain a database of DEMs for kettle holes using the SfM technique. The following conditions for obtaining data were adopted: (1) use of an entry-level digital single-lens reflex camera with a recording function (ground-based, hand-held technique), (2) scaling of the point cloud using simple field measurements based on the location of 4 up to 6 wooden stakes, (3) use of free software, mainly under the GUI license, (4) development of DEMs with a resolution of at least 0.05 m × 0.05 m, (5) use of an open repository for the prepared data set. The research aimed to simplify the measurements as much as possible and minimize costs while maintaining the high accuracy of the acquired data. After subsequent measurement seasons, the obtained material will be used as a reference to examine changes in the fresh relief of the landform in a short period (a few to several dozen months) and to calculate the components of the mass displacement balance within the kettle hole.

**Fig. 2:**
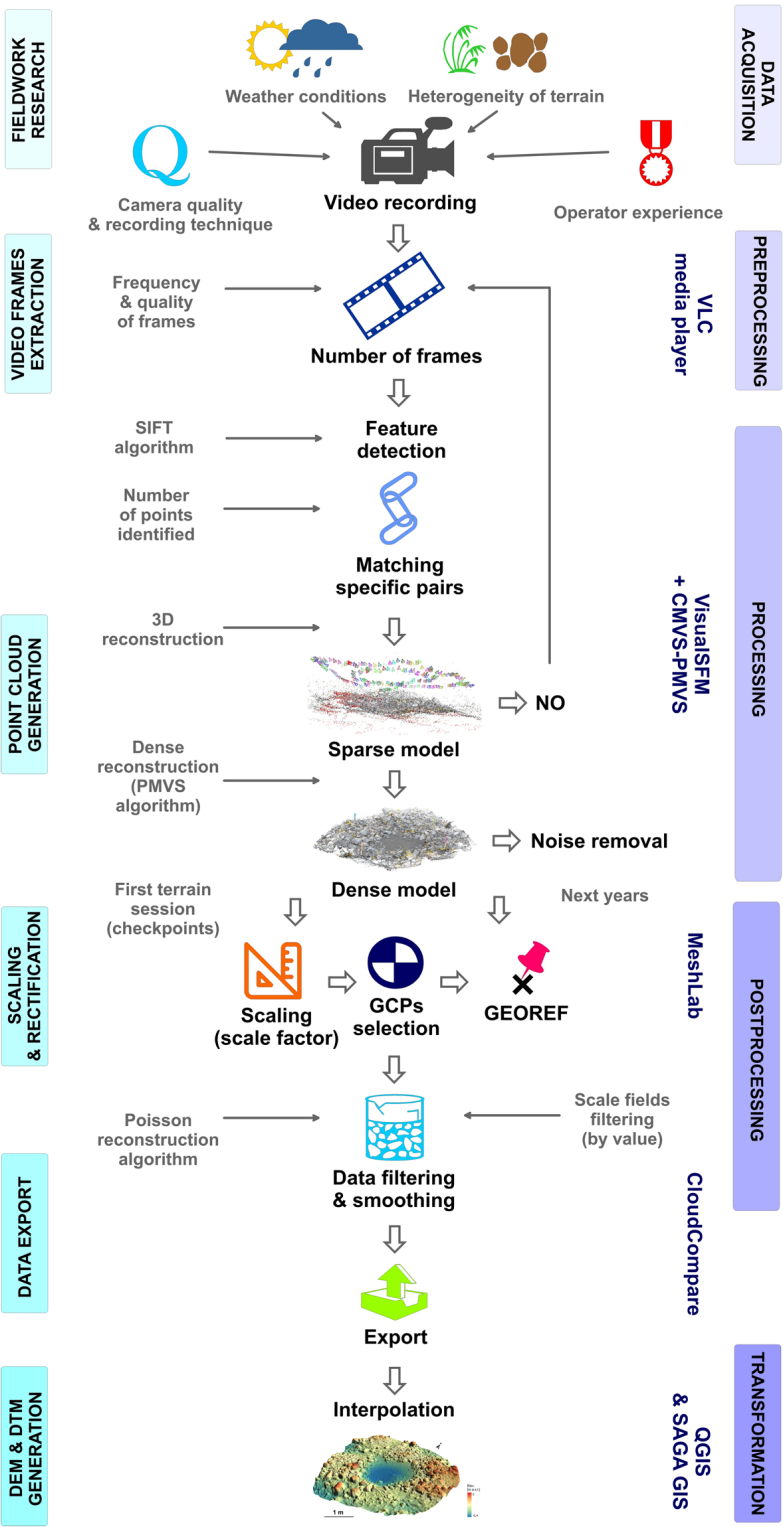
Scheme of data processing using open-source software.

Figure 2 presents a sequence of steps in obtaining DEMs. This process consisted of the following stages: (1) fieldwork research, (2) video frames extraction, (3) point cloud generation, (4) filtering, scaling and rectification, (5) data export, (6) and DTM generation. The method of processing video recordings into a dense point cloud and exporting data using the described open-source software was inspired by the publication of Wróżyński *et al*.^9^. The authors applied a low-cost SfM method for obtaining information about microtopography using a smartphone and a camera in laboratory and terrain conditions. They presented a procedure largely adopted in this study and described in detail in the **Methods** section (see also Fig. 2). This general scheme is also consistent with the typical workflow of Carrivick *et al*.^1^ (Fig. 3.1., p. 38).

## Methods

### Fieldwork research

**Fig. 3:**
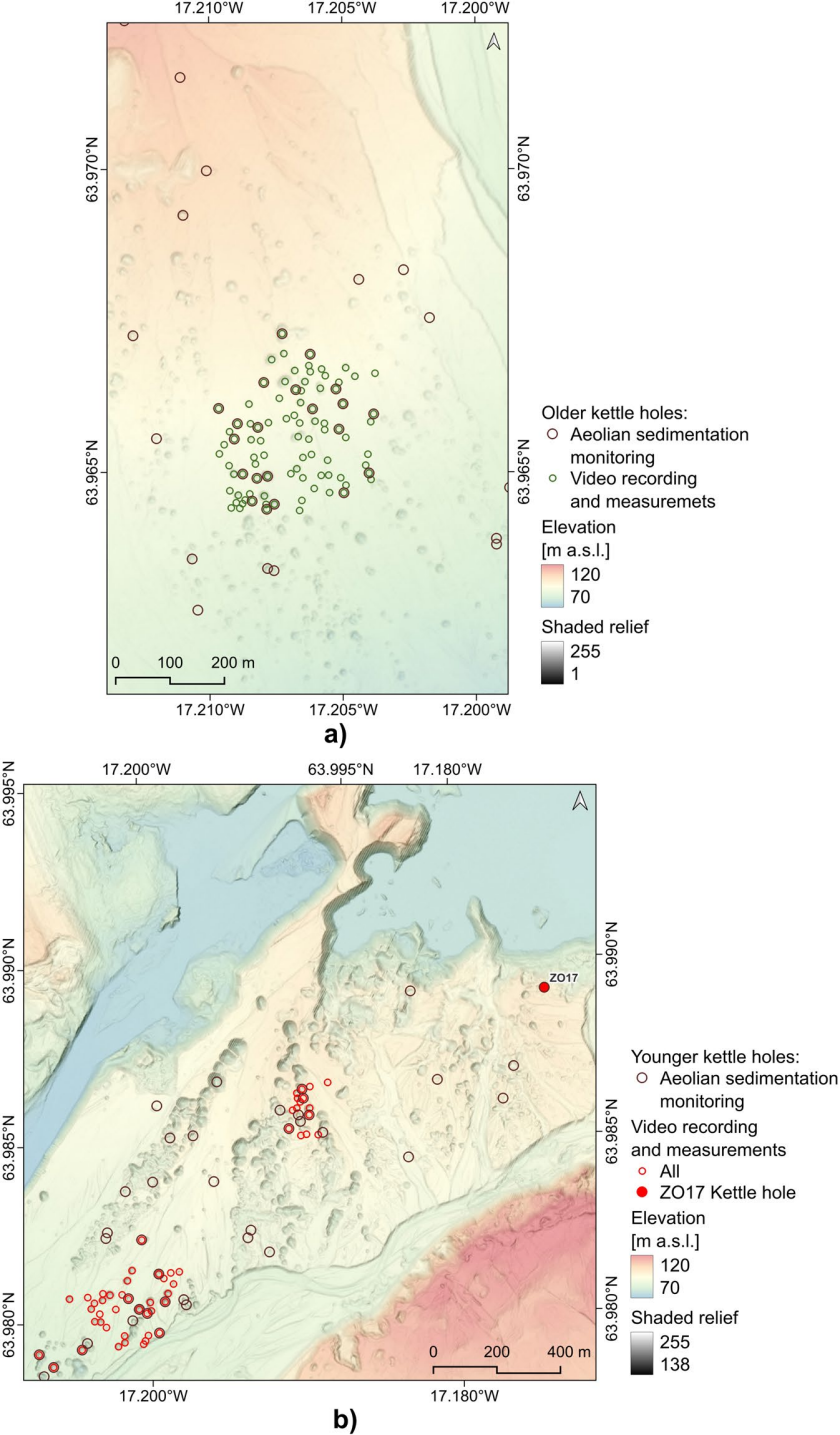
Location of kettle holes selected for filming on Skeiðarársandur in June 2022 (**a**) within the older sandur level and (**b**) within the younger level (based on ÍslandsDEM v. 1.0, 2 × 2 m, files 61 and 62, Landmælingar Íslands, https://dem.lmi.is/mapview/?application=DEM; 40% of the layer opacity). The ISN93/Lambert 1993 coordinate system is used (the WGS84 coordinates notation).

Data for the study were collected during a field session on Skeiðarársandur in southern Iceland in June 2022 and are freely accessible in the Zenodo repository^57,58^. Initially, 78 random kettle holes were selected to study the rate of aeolian sedimentation, and monitoring started in June 2021. The <Random Selection> option from <Research Tools> of vector layers in QGIS was used for this purpose (the <> signs indicate the name of an option/tool in a given program). Then, in 2022, clusters of kettle holes formed during several episodes of glacial floods (jökulhlaup) were selected in such a defined research area within two sandur levels. In the case of the first, older level, floods occurred during the maximum extent of Skeiðarárjökull glacier in the late 19th century and continued until the late 1930s^41,59^. The landforms are located in the proximal part of the sandur in the western part of the Haaldukvisl gorge (Fig. 3a; cf. Figure 1 and see also **Zenodo-Map_kettle-holes2022.png**^57^) at an altitude of approximately 87–96 m a.s.l. and a distance of approximately 4–5 km from the current position of the glacier front. Most have advanced vegetation cover, including single birch and willow trees. The second group consisted of younger-level landforms formed in the pathway of the catastrophic jökulhlaup of November 1996 (Fig. 3b; cf. Figure 1 and see also **Zenodo-Map_kettle-holes2022.png**^57^). They are located at an altitude of 85–100 m a.s.l., approximately 1.7–3 km from the glacier front. They are characterised by an initial degree of plant colonisation. All tested kettle holes were described spatially by centroid coordinates in ISN93, WGS84, and UTM coordinate systems (**Zenodo-KH_centroids_coord.zip**^57^).

Fieldwork began by installing 4–6 wooden stakes, 0.5 m long and 22 mm in diameter, around the edge of the kettle hole, measuring the height of the stakes (±1 mm) and the distance between them (±0.5 cm). The essential part of the work was video recording in the **MOV** format using a Nikon D3100 digital camera with a Complementary Metal Oxide Semiconductor (CMOS) sensor with a size of 14.2 million pixels and a focal length of 18 mm. Filming at a frequency of 24 frames per second consisted of circling the depression twice; the first time, the focus was on the stakes and the opposite edge of the landform, and the second time – on its interior. In this way, footage was obtained for 43 older landforms and 55 younger ones in 90 video files with a total size of approximately 50 GB^58^.

The average filming time was 253 s ± 135 s for the younger kettle holes and 193 s ± 76 s for the older level. It strictly depended on the landform size, i.e. twice its circumference, which had to be covered when filming the object. Another critical factor was the variety of terrain surfaces, affecting the comfort of the operator’s route; large boulders, narrow ridges between depressions, or willow and birch trees slowed down the rate of circling the landform. Due to the asymmetric distribution of the data, all average parameters reported are the median and the interquartile range (IQR). Calculations are available in the spreadsheet **Zenodo-Kettle-holes_parameters_2022.xls**^57^.

The stake position was not fitted into existing vertical and horizontal spatial reference systems. However, stakes were used to create local plane rectangular coordinate systems (values in meters) for each kettle hole to study changes over time. Moreover, the location of stakes contact with the ground was a network of checkpoints. Information about the stake parameters is available in the dataset in a ZIP file for each landform under the name **Zenodo-Number_stakes.txt**^57^.

### Video frames extraction

Further work was carried out using the software (Table 1). A Dell Precision 5530 laptop was used with an Intel® Core™ i9-8950HK CPU @ 2.90 GHz processor, 32GB RAM, an Intel® UHD Graphics 630/NVIDIA Quadro P2000 graphics card, and SSD drive.

**Table 1:** Software used to prepare data (access: March 15, 2024).

Software	Website	Licence Type
VLC media player ver. 3.0.19 (Vetinari)	https://www.videolan.org/	FREE, open source GNU GPL
VisualSFM – A Visual Structure from Motion System ver. 0.5.26	http://ccwu.me/vsfm/	FREE for personal, non-profit or academic use
CMVS/PMVS – Yasutaka Furukawa algorithm	https://www2.cs.sfu.ca/~furukawa/	GPL
MeshLab ver. 2021.05	https://www.meshlab.net/	FREE, open source GNU GPL
CloudCompare ver. 2.11.3 (Anoia)	https://www.cloudcompare.org/	FREE, open source GNU GPL
QGIS ver. 3.32 (Lima) and earlier versions	https://www.qgis.org/	FREE, open source GNU GPL
SAGA GIS ver. 9.1.2 and earlier versions	https://saga-gis.sourceforge.io/	FREE, open source GNU GPL

In VLC media player software, using <Scene filter> of the image tools, frames were extracted from the video at a frequency of 3–12 frames per second for stakes (to identify them in the point cloud) and one frame per second for the depression. Both sets of frames were then combined into one package. One frame had a size of 1,920 × 1,080 pixels and a resolution of 96 dpi in **JPG** format.

On average, 314 ± 24 frames per video were extracted, with a maximum of 334. With the given frame parameters, the maximum value resulted from the possibility of initiating the scene processing by another program, VisualSfM. Selected frames were included in the dataset (ZIP files for each kettle hole; **Zenodo-Number_frames** folder^57^).

### Point cloud generation

#### Sparse and dense models

**Fig. 4:**
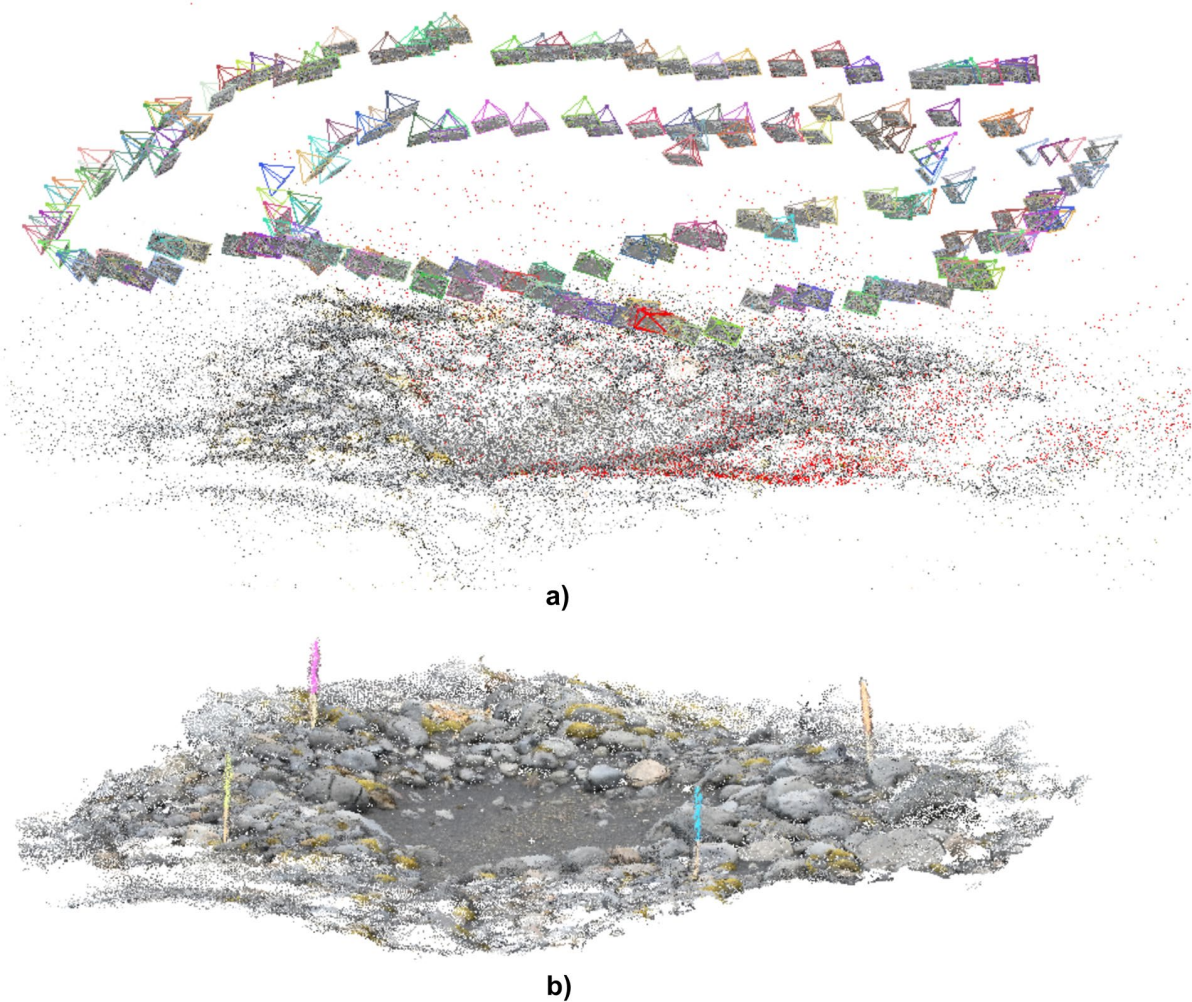
Point cloud obtained for the NZY15 kettle hole (an example) in VisualSFM and CMVS/PMVS: (**a**) sparse model with the visible camera position for selected video frames, (**b**) dense model – visible depression, boulders, vegetation and colourful stakes used for measurements.

In the next step, the VisualSFM program was used with the Clustering Views for Multi-view Stereo/Patch-based Multi-view Stereo Software (CMVS/PMVS) algorithm^9^. Changchang Wu developed VisualSFM, the fast-running application (multicore parallesim), for feature detection, feature matching, and bundle adjustment^60^. VisualSFM was, for example, used to monitor the position of a cliff in Ault in Northern France^61^. The author applied this program to create a point cloud based on 568 photographs and rectify the model to the Lambert-93 French official projection. Another application example would be monitoring the Super-Sauze landslide in the Southern French Alps using different surface reconstruction pipelines, including VisualSFM^62^. The free software PMVS was, in turn, used for dense reconstruction of the detritus dump located at Zijin Mine in Southeast China^63^ or to obtain a camera calibration certification (internal and exterior orientation), for example, for studying soil erosion in Tuscany, Italy^64^. <Compute Missing Pairwise Matches>, the first option, the most time-consuming stage, required an average of 155.5 ± 38 minutes to process the optimal number of frames per video with the previously specified laptop parameters. In this way, sufficient overlap in content was identified in the extracted frames (<Pairwise matching>) to find identical points to obtain a three-dimensional effect (<Compute 3D Reconstruction>). The program uses the scale-invariant feature transform (SIFT) algorithm to recognition of the key features. A point cloud was generated, obtaining a sparse model (Fig. 4a), and then, via the <Run Dense Reconstruction> option, a dense model, i.e. a dense point cloud with a texture (Fig. 4b). Noise was manually removed from the cloud, leaving only points directly related to a given depression. The point cloud was exported in **NVM** and **PLY** formats.

#### Point clouds

**Fig. 5:**
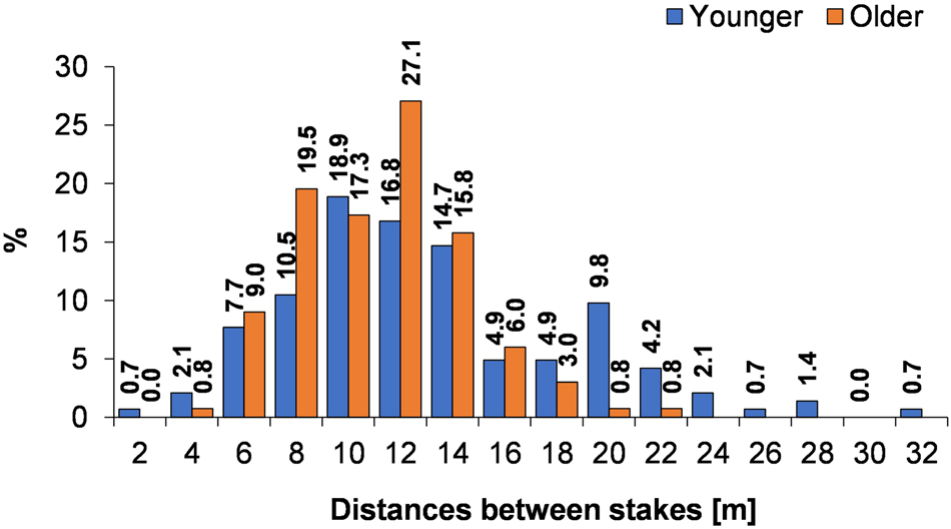
Distances between stakes in the filmed kettle holes in June 2022.

Point clouds were ultimately generated for 85 kettle holes. Those videos that were not recorded correctly in terms of the assumptions of the SfM technique were excluded from further analysis. Another cause was insufficient or no visibility of the stakes. In case 48 of the video, all measurement stakes were visible. A minimum of two adjacent stakes was necessary to scale the point cloud. The average distance between stakes was 11.8 m ± 6.1 m for younger landforms and 10.8 m ± 5 m for older ones. Half of the distance measurements in the case of younger landforms covered 8–14 m (Fig. 5), and the second smaller maximum appeared in the 18–20 m (almost 10%). For older depressions, the maximum was in the range of 10–12 m (over 27%), and almost 90% of the distances were in the range of 4–14 m. For larger landforms, the stakes were increased from 4 to 6. Hence, the histogram shows the type of saddle. Smaller distances between stakes made it easier to take measurements with a tape measure in stronger winds.

**Fig. 6:**
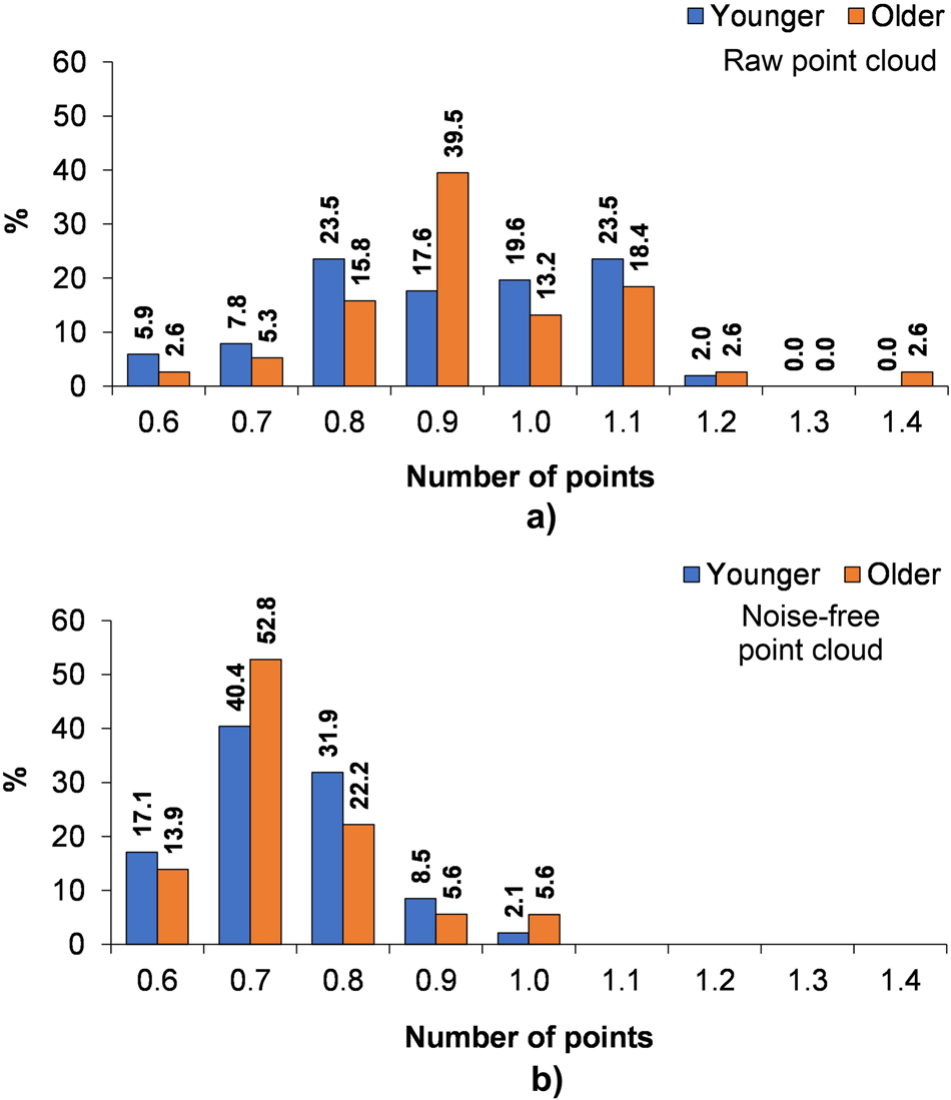
Number of points within the point cloud of the filmed kettle holes in June 2022: (**a**) raw point cloud, (**b**) noise-free point cloud.

As a result of the process, the raw point clouds had an average of 0.863 mln ±0.24 mln points for younger landforms and 0.865 mln ±0.19 mln points for older landforms. While the medians are similar, the data distribution differs (Fig. 6a). In the case of younger landforms, there are two dominants in the range of 0.7–0.8 mln and 1.1–1.2 mln points (23.5% of data each). It is mainly reflected in differences in vegetation cover and surfaces covered with fine-grained material. In the case of older landforms, almost 40% of the data is in the range of 0.8–0.9 mln points, and the depressions are more homogeneous in terms of surface coverage, mainly by vegetation (mosses, lichens, blueberry shrubs, heather, etc.).

In the rescaled point clouds, where the noise has been removed, the average values are 0.743 mln ±0.14 mln points for younger landforms and 0.733 mln ±0.1 mln points for older landforms, respectively (Fig. 6b). Both groups of landforms are characterised by the maximum of 0.6–0.7 mln points; 40.4% in the case of younger kettle holes and 52.8% for older forms. There are 4,061 points per 1 m^2^ of kettle hole, which averages approximately 10 points per area of 0.0025 m^2^, corresponding to the adopted resolution of the generated digital terrain models (0.05 × 0.05 m – one raster size). **PLY** files with scaled and noise-free point clouds were usually 15–40 MB and are available in the **Zenodo** dataset^57^ – in ZIP files for each kettle hole.

### Filtering, scaling and rectification

The next step was to scale the point cloud to the terrain dimensions obtained from field measurements. For this purpose, the <Transform: Scale, Normalize> tool from the <Normals, Curvatures and Orientation> filter in the MeshLab program was used^9^. Scaling was based on the scale factor, i.e. the ratio of the terrain distance to the distance read in the program using the <Measuring Tool>. Distance measurements were made when at least two adjacent stakes were visible in the point cloud, and stake height measurements were made when the entire stake was visible. The measurement was made ten times for each distance/height; the data was then averaged, and the scale factor was calculated. Training sessions were performed before the proper measurements were taken to familiarize with a given point cloud. All calculations were included in the dataset in spreadsheets (ZIP files for each kettle hole; **Zenodo-ErrorsNumber.xls/.xlsx** files^57^). Stake names (distance markings) come from the first two letters of the colour, e.g. OR means an orange stake.

Kettle holes are immersed in a relatively flat surface, with an average inclination of approximately 0.6–1.6°, where the lowest point of the depression is the minimum value of the elevation of later DEMs. In other cases, i.e. filming on the slope, it is necessary to obtain information about the surface slope to know the height differences between the bases of the stakes around a given landform. For this purpose, the slope can be determined based on other existing materials, or appropriate measurements can be made in the terrain.

Stakes distance and height measurements taken in the field allowed us to assess the accuracy of the data later used to create the DEM. It was the absolute value of the difference between the measured data and the data read (ten times) on the clouds before and after scaling (from now on referred to as the horizontal and vertical measurement error for simplicity). It corresponds to the values of root-mean-square error (RMSE). In the case of younger landforms, the average horizontal error (distance), taking into account the measurement error with a tape measure, was 0.032 m at IQR = 0.04 m, and the vertical error (height), taking into account the measurement error of the tool, was 0.004 m at IQR = 0.004 m. For older landforms, it was 0.022 m at IQR = 0.01 m and 0.002 m at IQR = 0.001 m, respectively. These error values corresponded to other reports when the handy-held, ground-based SfM technique was used^65,66^.

**Fig. 7:**
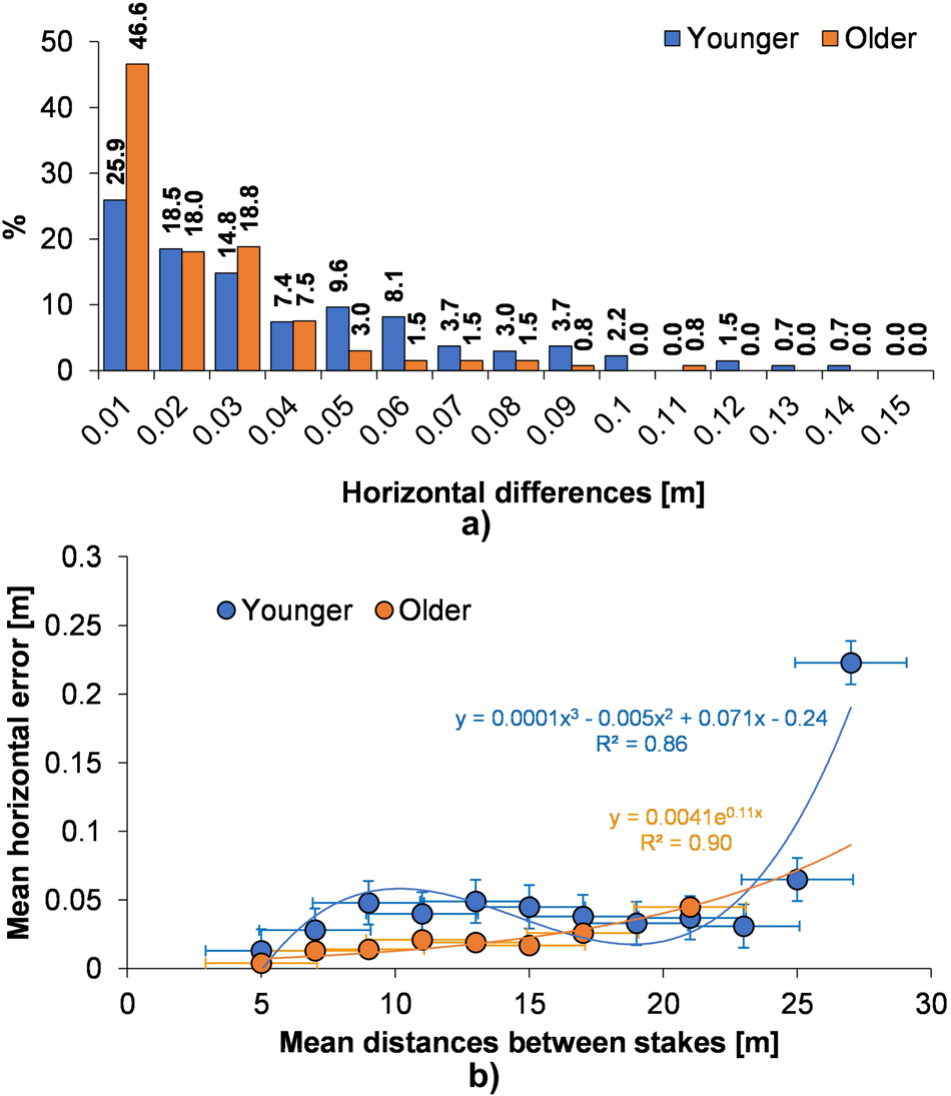
Statistical measures of kettle holes horizontal errors: (**a**) horizontal differences between the stakes distance measured during the fieldwork research and on point cloud in the software, (b) relationship between the mean stakes distances and mean horizontal errors (in groups of values arranged in ascending order) with standard error bars; p = 0.05, n = 12 for younger landforms and n = 8 for older kettle holes.

The vertical error remains relatively constant and low. It is because each 0.5 m long stake in the point cloud can be zoomed in as much as possible to read its height very clearly. The situation is different regarding the distance between stakes, which is about 20–30 times bigger. For both age groups, most differences in the horizontal error fall in the range up to 0.01 m – just over 25% of the measurements for younger landforms and almost half for older ones. Almost 85% are within the range of up to 0.06 m in the case of younger depressions, and almost 85% of the error values of older landforms are within the range of up to 0.03 m (Fig. 7a).

The comparison of the distances between stakes with the calculated horizontal errors shows direct relationships only for distances longer than 15 m (R = 0.65). After arranging the values of horizontal errors in ascending order, the average distances between stakes were calculated in each range of the calculated differences. This comparison indicates an 86–90% probability (Fig. 7b) that, according to this method, there is a statistically significant relationship (p = 0.05, n = 12 and n = 8) that up to 25 m of the distances between stakes horizontal errors should be smaller than 0.05 m on average. However, it is also visible that the horizontal errors rapidly increase above 25 meters of this distance.

In the case of kettle hole no. ZO17-2 within the younger sandur level, the point cloud was rectified to compare changes after the intervention in the relief of the landform bottom (the <GEOREF> option in MeshLab software). The model rectification process was based on reading local coordinates from characteristic points (stones), the so-called ground control points (GCP) from model ZO17-1, creating a GCP table and combining identical points of ZO17-2 model (the **Technical Validation** section).

### Data export

**Fig. 8:**
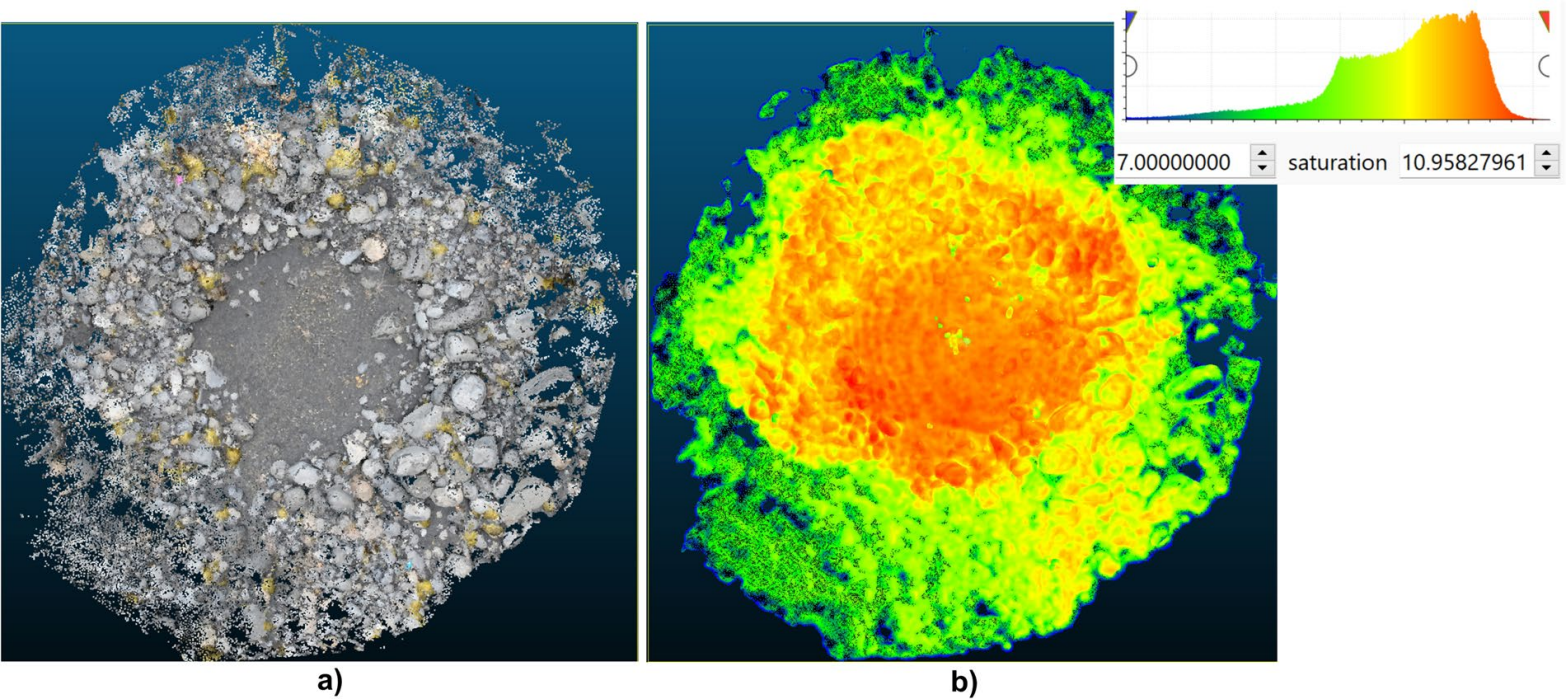
An example of a dense model of a NZY15 kettle hole in CloudCompare: (**a**) RGB model, (**b**) scalar fields model after smoothing with the Laplacian function and a point density histogram.

The scaled/rectified and denoised model in **PLY** format was imported to CloudCompare in the <Set Front View> option. We first see it as an RGB model (Fig. 8a). Information about the cloud density was obtained using the <Poisson Surface Reconstruction> plugin^67^. The main parameter, i.e. <octree depth >, was set to 10. Then, the <Scalar Fields> values obtained using the histogram (Fig. 8b) were applied to limit the cloud coverage to the target density from <Min> 7 to <Max> obtained from the <Filter By Value> option. Additionally, the data was smoothed by the <Laplacian> function with default settings of 20 iterations and a smoothing factor of 0.2. The resulting new mesh was exported in the **TXT** format, obtaining, among others, information about the X (first field) and Y coordinates (third field) and the Z height (second field).

### DEM generation

The generated text file was then used in geoinformation software like QGIS to process the data into a DEM. It required several additional operations such as (a) exporting the text data to the shapefile format, (b) saving Z as the third field and Y as the second one, (c) multiplying the Z column by the value –1 due to Z inversion^61^, (d) adding to the field Z the value of minimum Z – in this way, a local height system was obtained, where the minimum elevation of the depression in June 2022 is 0 m, (e) generating a mask of the kettle hole (a ZIP file for each landform; shapefile **Zenodo-Number_shapePoly**^57^), (f) removing model points located outside shape mask, (g) removing points from the model, related to stakes, artefacts and shadows of boulders causing a “pixelization” effect.

The obtained model was processed in two ways. The first involved re-converting the data to a **TXT** file with the DEM model (ZIP files for each landform; **Zenodo-Number_DEM.txt**^57^). The second approach generated digital terrain models (DTMs) in the SAGA GIS program. Using the <Gridding> tools, data was interpolated by the <Triangulation> method with a resolution optimal for the model (from 0.005 m × 0.005 m to 0.04 m × 0.04 m) and an assumed 0.05 m × 0.05 m for all depressions in the **GeoTIFF** format (ZIP files for each landform; **Zenodo-Number_TIN_resolution_w-stakes_blanked_norm.tif**^57^ and 3D view **Zenodo-Kettle-hole_Number.png**^57^).

Rescaling the model concerning the distance between the stakes allowed us to obtain local plane rectangular coordinate systems with the origin relative to the filming start point. Additionally, in the case of some models of the older sandur level, trees had to be manually removed. Hence, two files were the study result: one with the extracted place of the tree and the second one – with the reconstructed surface shape in the place of the tree using the <Fill nodata> option from the <GDAL Raster Analysis> tools in QGIS.

Vector geometry tools were used to calculate the Cartesian surface area of the kettle hole and its perimeter. Its maximum and average depth were also calculated, and using the <Grid Volume> tool in the SAGA GIS program – the volume of the landform.

## Data Records

The dataset is available at the international open repository Zenodo^57,58^ developed by the European OpenAIRE program under the Creative Commons Attribution 4.0 International license (CC BY 4.0). The data is organized in two parts: (I) – primary data in the form of point clouds, DEMs and DTMs of kettle holes (10.5281/zenodo.7449082^57^) and (II) – supplement to part I, original video files from the field session carried out in June 2022 on Skeiðarársandur (S Iceland) (10.5281/zenodo.7451375^58^). Part I contains, among others, files introducing the database (Table 2).

**Table 2:** Name and format of files describing the content of the first part of the dataset at the Zenodo repository^57^.

File	Content
ReadMe.txt	Title, DOI number, contact information, the title of the project under which the data was collected, description of the database structure, acknowledgements, link to part II by DOI number
Map_kettle-holes2022.png	Map of the study area
KH_centroids_coord.zip	A package of SHP, TXT and CSV files with kettle holes centroid coordinates in various coordinate systems
Kettle-holes_parameters_2022.xls	Main parameters of video files, point clouds and kettle holes
Documentation_2021-05-X-ST10-00710.pdf	Project documentation divided into sections: description of the purpose and context of the research, description of methods, database organization, acknowledgements, and references

The main contents are **ZIP** files. Each package characterises the kettle hole from the older (KH_JUNE2022_OLDER_NUMBER.zip) or younger levels of Skeiðarársandur (KH_JUNE2022_YOUNGER_NUMBER.zip). The database also includes four models obtained at the current lowest level (the Gígjukvísl river drainage pathway) in the inner marginal zone. The age and origin of the depressions are not known (KH_JUNE2022_YOUNGEST_NUMBER.zip). The contents of the packages are described in Table 3.

**Table 3:** The content of ZIP data packages for each kettle hole in part I of the dataset at the Zenodo repository^57^.

Folders/files	Content
NUMBER_frames folder	Frames extracted from video in JPG format
ErrorsNUMBER	Horizontal and vertical error calculation in XLS and XLSX formats
Kettle-hole_NUMBER	3D visualization of the kettle hole in PNG format – hypsometric tints and shaded relief
NUMBER_DEM	DEM in TXT format
NUMBER_model-w-noise.1	Scaled point cloud of the kettle hole, without noise, in PLY format
NUMBER_shapePoly	SHP file of kettle hole shape
NUMBER_stakes	Stakes description in TXT format – height and distances between stakes from terrain measurements
NUMBER_TIN_OPT-RES_w-stakes_blanked_norm	DTM of kettle hole of the optimal resolution in GeoTIFF format
NUMBER_TIN_0i05m_w-stakes_blanked_norm	DTM of kettle hole of the 0.05 × 0.05 m resolution in GeoTIFF format

Part II contains the ReadMe.txt file, with the same content as in Part I and **MOV** files for each landform.

## Technical Validation

The most natural data validation obtained using the SfM technique is material from terrestrial laser scanning (TLS)^1^. It is due to creating non-selectively sampled data as a point cloud. The author does not have such data for this area or other data from classical photogrammetric techniques or differential Global Positioning System (dGPS) measurements. The DEMs available on the National Land Survey of Iceland website from the Airborne Laser Scanner (ALS) have a 2 × 2 m resolution. Since the data were not rectified to existing topographic coordinate systems, validation was based mainly on field measurements of the distances between stakes arranged around the landform and their height. On this basis, the vertical and horizontal errors of the point clouds were calculated, which is described in detail in the **Methods** – the **Filtering, scaling and rectification** section. The data set in the Zenodo repository includes the calculations (ZIP files for each kettle hole; **Zenodo-ErrorsNumber.xls/.xlsx** files^57^).

**Fig. 9:**
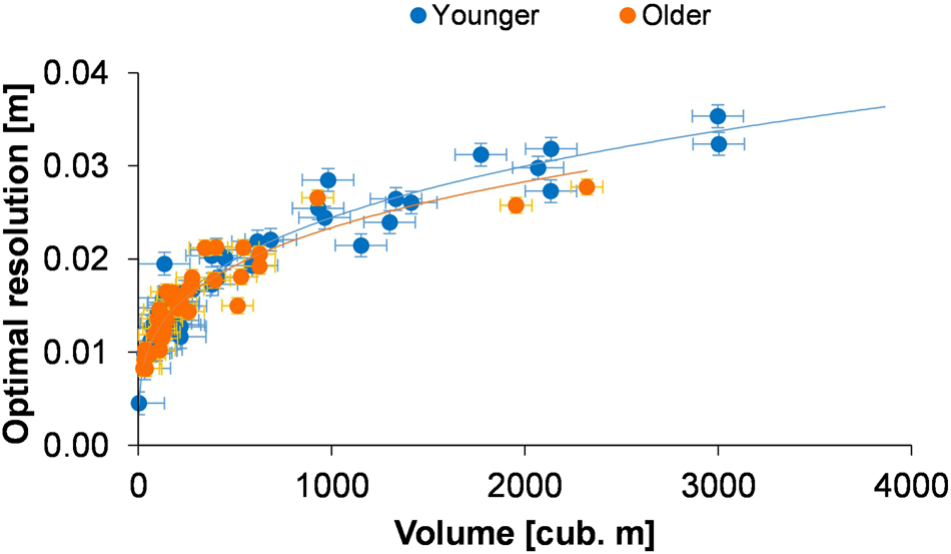
The relationship between the volume of kettle holes and the optimal resolution of DEMs with standard error bars.

The method of processing points set into DEM (and DTM) was used in proven, popular open-source geoinformation programs such as QGIS and SAGA GIS (see Table 1 and the **Code Availability** section). For interpolation purposes, the commonly used Delaunay Triangulation method was adopted^68,69^. The optimal resolution of the DEM was determined based on the density of points falling on a given kettle hole. The solution considers the raster size so that statistically, at least 1 point is located within its area. Creating a DEM based on the SfM technique with the described camera parameters allowed us to obtain an optimal resolution of up to 0.04 × 0.04 m for depressions with a volume of up to 4,000 m^3^ (Fig. 9). For larger kettle holes, the optimal resolution will decrease with the power function.

The methodological repeatability was checked on the example of the ZO17 kettle hole (see Fig. 3b) based on the same set of 4 stakes (Table 4). The depression was filmed twice in 2022, first without intervening in the relief and then after trying to find a sedimentation monitoring stake from 2021 buried by the debris flow at the bottom. It was assumed that the generated point clouds should be the same in intact parts.

**Table 4:** ZO17 stakes position in local horizontal and vertical coordinate systems (in metres).

Stakes	X_loc_	Y_loc_	Z_loc_
Red (RE)	11.465	6.951	1.885
Yellow (YE)	17.018	2.683	2.907
Pink (PI)	11.880	−4.776	2.555
Orange (OR)	3.567	−1.715	2.711

**Fig. 10:**
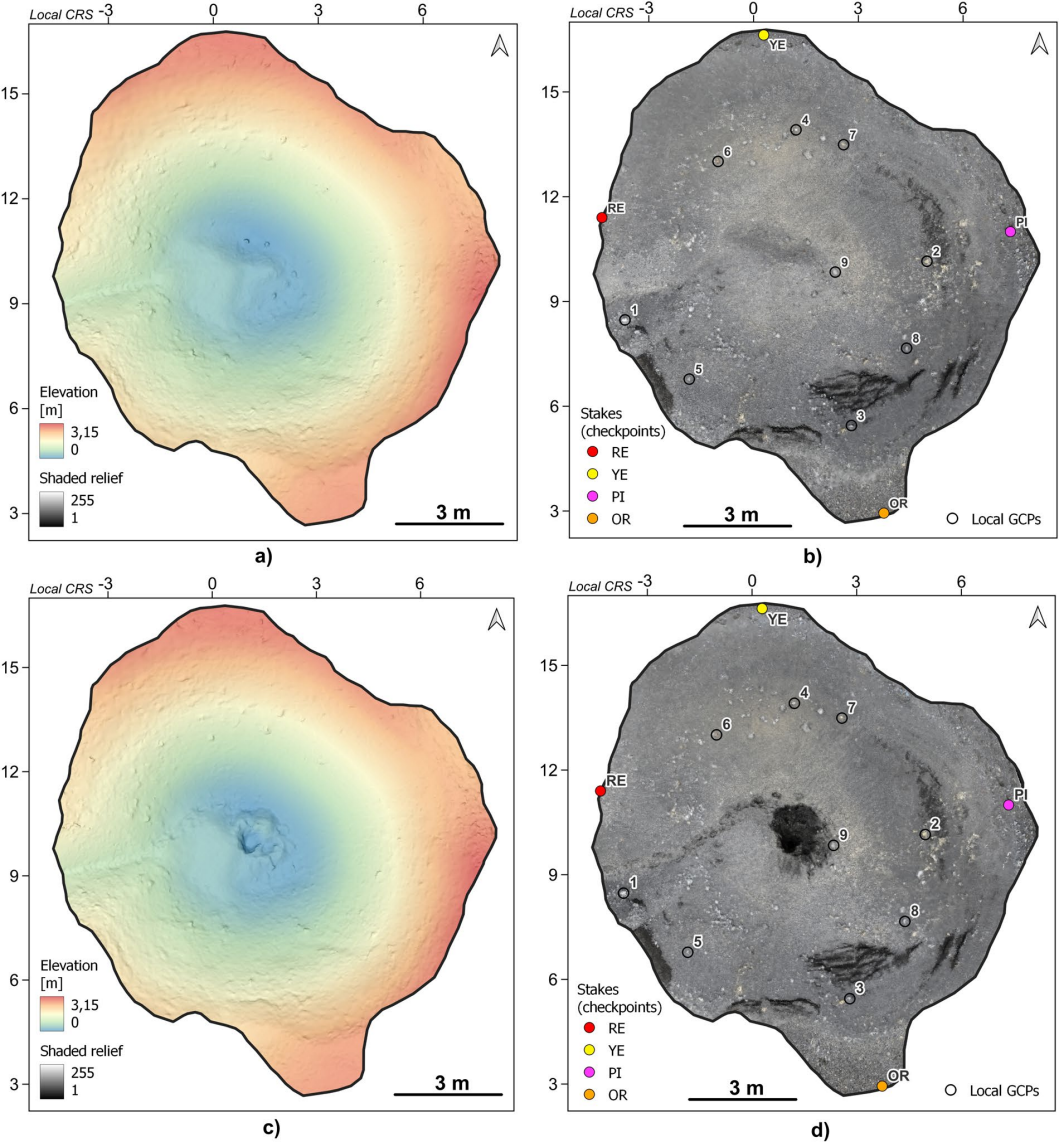
Control of methodological correctness of the kettle hole no. ZO17 for the 2022 DEM: (**a**) original landform relief (ZO17-1), (**b**) original kettle cover as an RGB model with the position of checkpoints (stakes) and GCPs, (**c**) landform relief after digging the bottom (ZO17-2), (**d**) kettle cover as an RGB model after bottom relief changes with the position of checkpoints and GCPs. The local horizontal and vertical coordinate systems are used (the coordinate notation in metres).

The first point cloud (ZO17-1) was scaled using field measurements (Fig. 10a,b; and **Zenodo-KH_JUNE2022_YOUNGER_ZO17-1.ZIP**^57^). The second point cloud (ZO17-2) (Fig. 10c,d; and **Zenodo-KH_JUNE2022_YOUNGER_ZO17-2.ZIP**^57^), after changes in the relief, was rectified based on nine control points in local coordinates (Table 5; see Fig. 10b,d). The average residual error in the rectification process was 0.012 m ± 0.006 m.

**Table 5:** Local GCPs (pebbles) of the ZO17 kettle hole with modified relief with the residual error values used to rectify the point cloud to check methodological repeatability.

Point	X _(ref ZO17-1)_	Y _(ref ZO17-1)_	Z _(ref ZO17-1)_	X _(picked ZO17-2)_	Y _(picked ZO17-2)_	Z _(picked ZO17-2)_	Residual Errors
1	8.582	6.086	1.3349	4.922	2.536	−1.7348	0.0145
2	10.866	−2.445	1.4743	11.320	2.228	4.3635	0.0200
3	6.010	−0.612	1.8539	12.047	1.926	−0.8002	0.0090
4	14.367	1.569	1.6458	6.125	2.073	5.4999	0.0067
5	7.014	4.127	1.3818	7.406	2.484	−2.1865	0.0072
6	13.305	3.738	1.2943	4.746	2.484	3.5165	0.0230
7	14.038	0.176	1.6981	7.513	2.007	5.8688	0.0112
8	8.334	−2.036	1.3210	12.189	2.410	1.9208	0.0099
9	10.372	0.160	0.1461	9.287	3.611	2.6906	0.0088
						Mean	0.0122

**Fig. 11:**
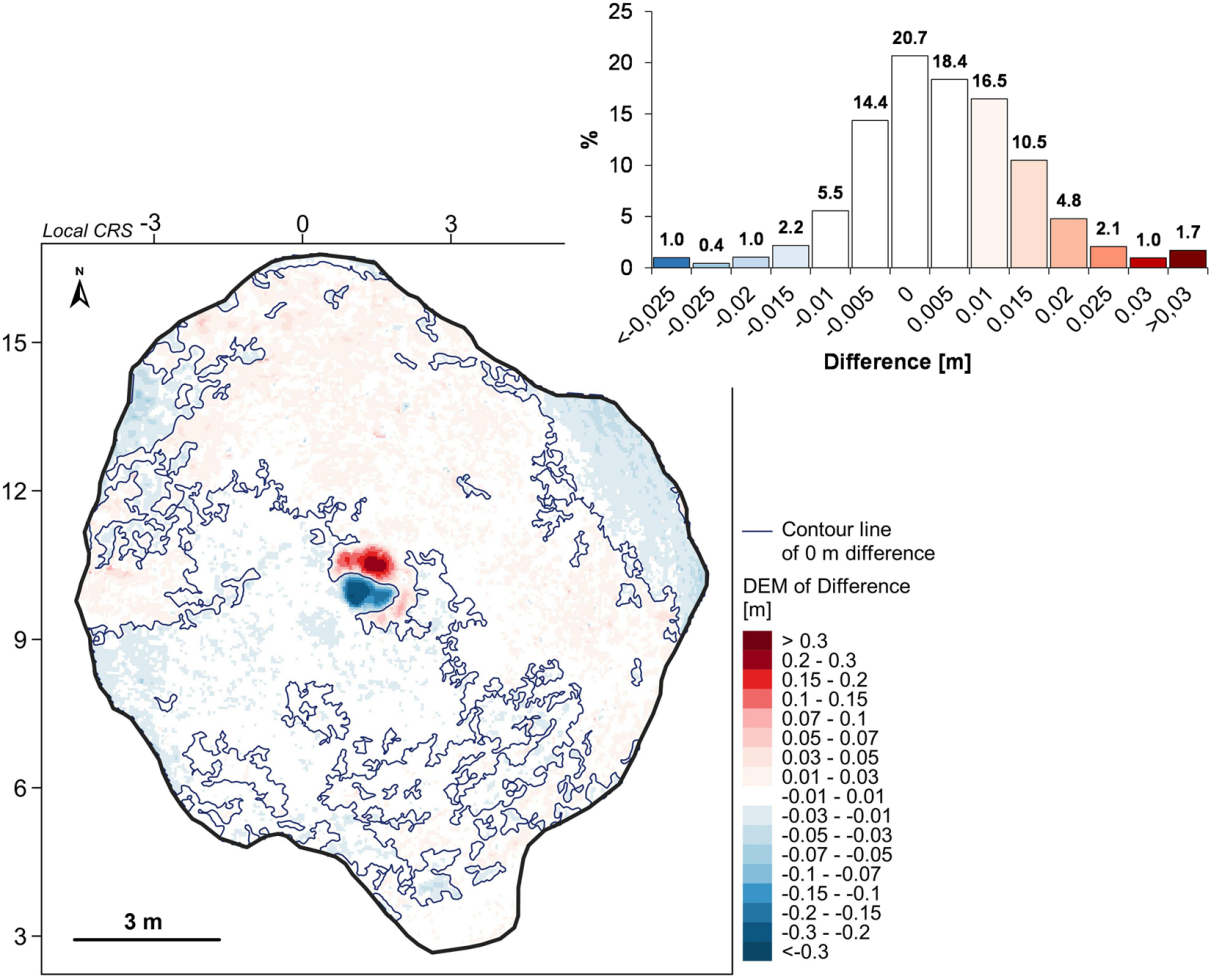
Control of methodological correctness – DEM of Difference between the original relief of the kettle hole (ZO17-1) and the modified relief (ZO17-2) and histogram, younger Skeiðarársandur level, June 2022. The local horizontal and vertical coordinate systems are used (the coordinate notation in metres).

Based on the heights of the stakes and the distance between them, the horizontal and vertical error of the point cloud after scaling (**Zenodo-ErrorsZO17-1.xls/xlsx**^57^) and after rectification (for ZO17-2; Table 6) was also calculated. For stake heights, the scaling error was from 0.003 to 0.01 m (average 0.006 m), and the rectification error was from 0.001 to 0.007 m (average 0.004 m). For the distance between stakes, the horizontal error is from 0.005 to 0.028 m for scaling (average 0.015 m) and from 0.001 to 0.093 m for rectification (average 0.031 m). The adopted target resolution of 0.05 × 0.05 m for the DEM should be optimal to examine the evolution of the relief of kettle holes. It is also indicated by comparing both DEMs in the form of the DEM of Difference (DoD) model (Fig. 11). Scaling and rectification errors cause height differences up to ±0.01 m to cover almost 60% of the depression area and differences up to ±0.02 m to almost 90% of the area—more significant differences concern only the place of the relief intervention in the landform bottom.

**Table 6:** Measurements (M) of the height (h) of stakes and the distance (d) between them on the rectified point cloud ZO17-2 in the MeshLab program and averaged error values (in metres).

Parameter	OR (h)	PI (h)	YE (h)	OR–PI (d)	PI–YE (d)	YE–RE (d)	RE–OR (d)
M1	0.336	0.323	0.353	8.857	8.950	7.093	11.733
M2	0.340	0.326	0.354	8.849	9.053	7.107	11.731
M3	0.339	0.327	0.351	8.855	8.955	7.116	11.720
M4	0.340	0.324	0.354	8.852	8.966	7.118	11.742
M5	0.342	0.328	0.353	8.856	9.055	7.114	11.722
M6	0.340	0.325	0.353	8.848	9.056	7.118	11.739
M7	0.339	0.329	0.352	8.856	8.962	7.114	11.739
M8	0.342	0.328	0.354	8.856	8.961	7.118	11.727
M9	0.340	0.324	0.352	8.854	8.968	7.114	11.746
M10	0.342	0.327	0.353	8.857	9.051	7.120	11.748
Mean [m]	0.340	0.326	0.353	8.854	8.998	7.113	11.735
Median [m]	0.340	0.327	0.353	8.855	8.967	7.115	11.736
Terrain value [m]	0.345	0.320	0.354	8.840	9.060	7.130	11.735
Difference [m]	0.005	0.007	0.001	0.015	0.093	0.015	0.001
Av. errors [m]		h _mean_ =	0.004			d _mean_ =	0.031

## Data Availability

No custom code was used during this study for the curation and/or validation of the dataset. Table 1 in the **Methods** section presents the list of programs used in data processing. VLC media player ver. 3.0.19 (Vetinari) extracted frames of a given frequency (recording ratio) from video files. “VLC is a free and open source media player, encoder and streamer created by the VideoLAN volunteer community”^70^. A program by Changchang Wu, VisualSFM – A Visual Structure from Motion System, ver. 0.5.26 was used to process the film frames into a sparse point cloud. “VisualSFM is a GUI application, free for personal, non-profit or academic use for 3D reconstruction using structure from motion”^71^. Using the Yasutaka Furukawa Clustering Views for Multi-view Stereo/Patch-based Multi-view Stereo Software (PMVS/CMVS) algorithm^72^ together with VisualSFM enables the generation of a dense point cloud. The program is distributed under the GPL license. Scaling and rectification of the dense model was carried out in MeshLab ver. 2021.05^73^. It is a free, open-source application under the GNU GPL license “for processing and editing 3D triangular meshes”^74^. The filtered data was exported to a text file in another free, open-source program under the GNU GPL license, CloudCompare ver. 2.11.3 (Anoia). It is used for 3D point cloud and mesh processing^75^. Creating DEMs and DTMs, as well as visualizations in the form of maps, was carried out in popular free open-source geoinformation programs under the GNU GPL license, such as QGIS ver. 3.32 Lima and earlier versions^76^ and SAGA GIS ver. 9.1.2 and earlier versions^77^.
